# Prognostic Value of a Composite Inflammation–Renal Function Score in Type A Aortic Dissection

**DOI:** 10.3390/jcdd13030133

**Published:** 2026-03-11

**Authors:** Rui-Qin Zhou, Yin-Ding Peng, Hao Cai, Cheng Zhang, Qing-Chen Wu

**Affiliations:** Department of Cardiothoracic Surgery, The First Affiliated Hospital of Chongqing Medical University, Chongqing 400016, China; 204066@hospital.cqmu.edu.cn (R.-Q.Z.); 2025110298@stu.cqmu.edu.cn (Y.-D.P.); 2024140131@stu.cqmu.edu.cn (H.C.);

**Keywords:** type A aortic dissection, prognosis, systemic inflammation response index, serum creatinine, nomogram

## Abstract

Background and Objectives: Systemic inflammation and renal dysfunction play a central role in the progression and prognosis of type A aortic dissection (TAAD). This study evaluated the SCr score, a composite index combining the systemic inflammation response index (SIRI) and serum creatinine, to assess its prognostic value postoperatively. Materials and Methods: Clinical data from 299 surgically treated TAAD patients were retrospectively analyzed. SCr scores were stratified into three levels using optimal cutoffs. Survival differences were examined using Kaplan–Meier curves. Independent predictors of overall survival (OS) and in-hospital mortality (IHM) were identified through multivariable Cox and logistic regression, respectively. A prognostic nomogram integrating SCr and significant clinical variables was developed, and model performance was evaluated and compared with previously published models. Results: Higher SCr scores were associated with a progressively increased mortality risk. In multivariable Cox analysis, both SCr scores of 1 and 2 emerged as independent predictors of worse long-term survival, with SCr = 2 demonstrating a particularly strong association (hazard ratio (HR) = 4.408, 95% confidence interval (CI): 1.786–10.881; *p* = 0.001). In logistic regression analysis, SCr scores remained an independent predictor of IHM (SCr = 1: odds ratio (OR) = 3.066, 95% CI: 1.032–9.102; SCr = 2: OR = 4.811, 95% CI: 1.081–21.409; *p* < 0.05 for both). A prognostic nomogram based on the SCr score and other clinical variables achieved strong discrimination for OS (area under the curve [AUC]: 0.845) and IHM (AUC: 0.821). Conclusions: The SCr score was independently associated with preoperative risk in patients with TAAD. An SCr-incorporating nomogram demonstrated favorable discriminative performance for predicting overall survival and in-hospital mortality. These findings suggest that SCr-based assessment may provide complementary information and assist in the identification of high-risk patients within established clinical assessment frameworks.

## 1. Introduction

Type A aortic dissection (TAAD) is a rapidly progressive aortic emergency marked by hemodynamic instability, organ malperfusion, and high early mortality. Death rates rise sharply within hours of symptom onset, and even with timely surgical intervention, 30-day mortality remains close to 50% [1]. Therefore, effective early risk stratification is essential. Inflammatory activation contributes substantially to TAAD progression. Aortic wall injury initiates leukocyte recruitment, cytokine release, and coagulation cascade activation, which facilitate medial degeneration and extension of the dissection flap [2]. Correspondingly, inflammation-related biomarkers such as C-reactive protein (CRP), procalcitonin (PCT), interleukin-6 (IL-6), neutrophil-to-lymphocyte ratios (NLR), and platelet-to-lymphocyte-ratio (PLR) have been associated with adverse short-term outcomes [3,4,5]. However, conventional markers provide a limited perspective, potentially overlooking the systemic immune and hematologic perturbations characteristic of TAAD, underscoring the need for more integrated inflammatory indicators.

The systemic inflammation response index (SIRI), derived from neutrophil, monocyte, and lymphocyte counts, provides a composite measure of immune activation and myeloid-lymphoid balance. Although emerging evidence links the SIRI to cardiovascular risk and multi-organ stress, its prognostic value in TAAD, particularly for long-term survival, remains insufficiently defined [6,7,8].

Systemic inflammation and hemodynamic compromise in TAAD also predispose patients to renal injury. Experimental and clinical data suggest that the kidney is highly vulnerable to inflammatory and ischemic insults, and elevated serum creatinine levels often reflect both malperfusion and inflammation-mediated organ damage. Accordingly, research findings demonstrate that elevated serum creatinine levels correlate with a higher incidence of postoperative complications and mortality in TAAD patients [9,10,11,12,13,14].

As the SIRI reflects systemic inflammatory burden and serum creatinine indicates inflammation-related renal dysfunction, combining these markers may yield a more comprehensive assessment of disease severity. Accordingly, this study aimed to evaluate the prognostic utility of integrating preoperative SIRI and serum creatinine for predicting clinical outcomes in TAAD patients.

## 2. Materials and Methods

**Figure 1:**
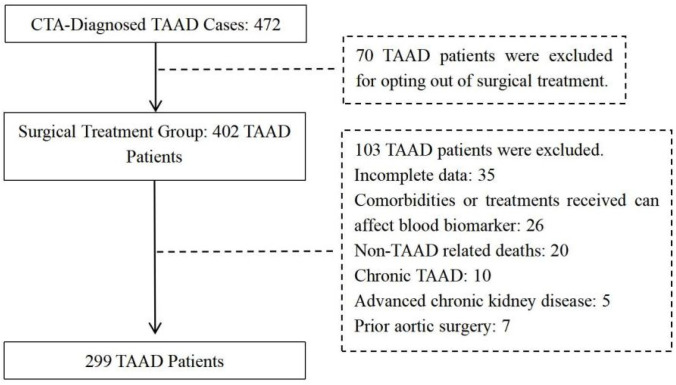
Workflow for patient inclusion.

This retrospective cohort study aimed to investigate the association between clinically relevant indicators and survival outcomes in patients with acute aortic dissection undergoing open surgical repair. Patients receiving surgical intervention for Stanford type A aortic dissection at the Department of Cardiothoracic Surgery, the First Affiliated Hospital of Chongqing Medical University, were retrospectively reviewed over a 6-year period (January 2015 to January 2021). All surgical procedures were performed according to institutional standards by experienced cardiovascular surgeons. Patients who met the following inclusion criteria were enrolled: (1) age range of 18–75 years; (2) a diagnosis of acute Stanford type A aortic dissection, as confirmed by computed tomography angiography (CTA) scan; and (3) having undergone a combined surgical procedure consisting of proximal aortic/root reconstruction along with total aortic arch replacement and frozen elephant trunk implantation. Patients were excluded based on the following criteria: (1) hematologic disorders, active infections, autoimmune/inflammatory diseases, or use of medications affecting inflammatory or renal biomarkers (e.g., corticosteroids, immunosuppressants, and renal replacement therapy); (2) advanced chronic kidney disease (stage 4–5) or long-term dialysis; (3) more than one thoracotomy, prior aortic surgery or known genetic aortopathies (e.g., Marfan or Loeys–Dietz syndrome); or (4) death unrelated to TAAD (e.g., trauma, malignancy, or non-cardiovascular causes). A total of 299 patients with confirmed TAAD were enrolled in the final analysis (Figure 1).

### 2.1. Data Collection

Basic information of 299 patients was collected retrospectively from electronic medical records at our institution. Demographic data included gender and age, and risk factors included body mass index (BMI), history of smoking and alcohol use, hypertension (HTN), and diabetes mellitus (DM). The patient’s status at admission comprised heart rate, blood pressure, the presence or absence of shock, pain, altered consciousness, pericardial effusion, and pleural effusion. Preoperative laboratory results encompassed parameters from routine blood tests, liver function tests, renal function tests, coagulation profile, and cardiac biomarkers. Intraoperative conditions, including perfusion times (operation, cardiopulmonary bypass [CPB], and aortic cross-clamp [ACC]), together with blood loss and transfusion volume were recorded. All patients in this study underwent total arch replacement under a standardized deep hypothermic circulatory arrest protocol, with target core temperatures maintained between 26 °C and 28 °C during the critical phase of cerebral and systemic protection. Survival data were obtained from the medical record system and supplemented by telephone follow-up.

Short-term outcomes were defined as in-hospital mortality (IHM), and long-term prognosis was assessed using overall survival (OS), which was measured from the date of surgery until death or the last follow-up. The primary study outcome for this cohort was TAAD-related death.

### 2.2. Optimal Cut-Off Values

Determination of optimal cut-off values, including age, SIRI, blood urea nitrogen (BUN), creatine kinase-muscle/brain (CK-MB), lactate dehydrogenase (LDH), serum creatinine, CPB time, intraoperative blood loss volume, and onset-to-surgery time was performed with X-tile software (version 3.6.1). X-tile applies a minimum *p*-value approach based on log-rank statistics across all potential thresholds, with internal correction for multiple testing, thereby providing an objective and reproducible method for identifying prognostically relevant cutoffs [15]. In our cohort, the optimal cut-off values were identified for age (53 years), SIRI (5.9), BUN (18.3 mmol/L), CK-MB (8.6 μg/L), LDH (470 U/L), serum creatinine (138 μmol/L), CPB time (321 min), intraoperative blood loss (780 mL), and onset-to-surgery time (6 h) (Figure S1A–I).

### 2.3. Definition of the Composite Inflammation-Renal Function Score

The composite inflammation–renal function score, termed the SIRI–Creatinine composite index (SCr), was derived from both the systemic immune inflammation index (SIRI) and serum creatinine levels. The stepwise scoring system was adopted to reflect the cumulative biological burden of systemic inflammation and renal dysfunction, with their coexistence signifying a more advanced disease state. Compared with weighted or continuous models, this simple ordinal approach helps reduce collinearity, mitigates overfitting in multivariable models, and improves clinical applicability by offering an intuitive, bedside-friendly stratification. While dichotomization of continuous variables may result in some information loss, the stepwise scheme provides a practical balance between statistical efficiency and interpretability. The validity of such additive, ordinal composites has been supported by prior prognostic tools, such as the modified Glasgow Prognostic Score, which has demonstrated robust utility across diverse patient populations [16].

Accordingly, the SIRI was calculated as (neutrophil count × monocyte count)/lymphocyte count. The determination of optimal cutoff values for the SIRI (cutoff: 5.9) and serum creatinine (cutoff: 138 μmol/L) was performed with X-tile software, which identifies thresholds that maximize the log-rank χ^2^ statistic for survival differences based on OS. Both variables were dichotomized into high and low groups accordingly. Patients were stratified according to the SIRI and serum creatinine levels into three groups: a score of 0 for low in both, 1 for high in either, and 2 for high in both (Table 1). The resulting SCr score was modeled as an ordinal categorical variable, and it was analyzed using univariate and multivariate regression to determine its independent association with both short-term and long-term outcomes.

### 2.4. Variable Selection and Multicollinearity Assessment

Variables included in the multivariable regression model were selected based on univariable screening and clinical relevance. Specifically, candidate variables were identified if they demonstrated an association with the outcome in univariable analyses and were deemed clinically meaningful according to prior literature and expert judgment. Accordingly, the final multivariable model comprised age, shock, SCr category, blood loss, CPB time, pleural effusion, BUN, CK-MB, LDH, renal artery involvement on CTA, stroke, surgical approach, and onset-to-surgery time. Notably, SIRI and serum creatinine were not modeled as independent covariates, given that both measures were incorporated into the composite SCr category, thereby reducing redundancy among correlated predictors and limiting the risk of multicollinearity.

Potential multicollinearity among the included covariates was formally assessed using variance inflation factors (VIFs). All variables exhibited low VIF values (range: 1.00–2.00), indicating the absence of clinically relevant multicollinearity (Table S1).

### 2.5. Statistical Analysis

Student’s *t*-test or Mann–Whitney U test was used to compare continuous variables, which are reported as mean ± standard deviation if normally distributed, or as median and interquartile range otherwise. The Chi-square or Fisher’s exact test was used to compare categorical variables, which are reported as frequency (percentage). Determination of optimal cutoff values (age, SIRI, BUN, CK-MB, LDH, serum creatinine, CPB time, and intraoperative blood loss) was performed with X-tile software (Yale University, New Haven, CT, USA). Assessment of correlations between variables was performed with Pearson’s chi-square test, with the correlation strengths among the blood parameters depicted using a heatmap. Survival curves were constructed based on Kaplan–Meier analysis. Survival predictors were quantified using Cox regression, with results expressed as hazard ratios (HRs) with 95% confidence intervals (CIs). Corresponding odds ratios (ORs) with 95% CIs were derived from logistic regression. Receiver operating characteristic (ROC) curves and area under the curve (AUC) values were used to assess the prognostic value of indicators and models. A two-sided *p* value of <0.05 was considered statistically significant. All analyses were performed with R version 4.4.1 (R Foundation for Statistical Computing, Vienna, Austria) and SPSS version 27.0 (IBM, Chicago, IL, USA), and graphical presentations were generated with GraphPad Prism version 9 (GraphPad Software, San Diego, CA, USA).

## 3. Results

### 3.1. Patient Characteristics

A total of 299 patients were included in the final analysis. Most participants were male (223/299, 74.6%), and the average age was 50.2 ± 9.2 years. During follow-up, 68 patients (22.7%) died and were classified as non-survivors, with the remaining 231 patients comprising the survivor group. Table 2 presents a descriptive summary of baseline and perioperative variables stratified by survival status. Numerically higher preoperative white blood cell counts and levels of serum creatinine, BUN, LDH, and CK-MB were observed in the non-survivor group. In addition, numerically longer operation time, CPB time, and ACC time, along with numerically greater intraoperative blood loss and red blood cell transfusion volumes, were also recorded in this group.

Hypertension was the most common comorbidity, present in 205 patients (68.6%). Among non-survivors, 47 patients (69.1%) had a history of hypertension, similar to that observed among survivors (68.4%). Abdominal pain at admission was relatively uncommon (24 patients, 8.0%). Among these patients, 18 died (75%) during follow-up. Based on preoperative imaging, all patients were classified as having DeBakey type I aortic dissections; no type II cases were identified. Additionally, renal artery involvement on CTA was present in 70 patients (23.4%). Regarding surgical details, the mean onset-to-surgery time was 24.8 ± 23.4 h in non-survivors and 16.5 ± 12.3 h in survivors, and in both groups the Bentall procedure was the most common approach for proximal aortic/root reconstruction, followed by the Wheat procedure and isolated ascending aortic replacement.

### 3.2. Correlation Among Seven Clinically Relevant Parameters

**Figure 2:**
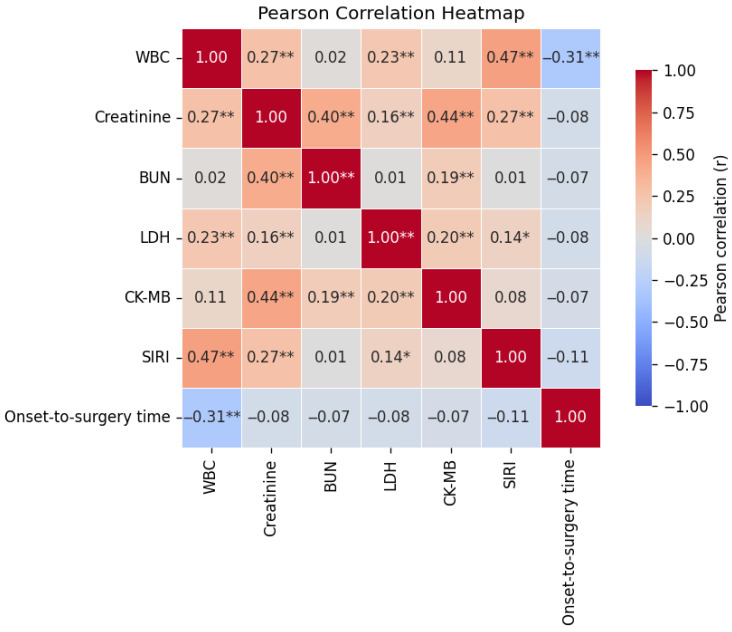
Correlation heatmap of seven clinically relevant parameters. Notes: * denotes *p* < 0.05, ** denotes *p* < 0.01.

The heatmap displays the strengths of the correlations among the seven clinically relevant parameters (Figure 2). Warmer and deeper red hues correspond to stronger positive correlations, with the spectrum spanning from light pink (weak) to dark red (strong). The exact Pearson correlation coefficient (r) value within each cell indicates the linear association between two variables: ±1 denotes a perfect positive or negative correlation, whereas 0 indicates no linear association. Statistical significance levels are explicitly marked: * for *p* < 0.05 and ** for *p* < 0.01.

Pearson’s correlation analysis was conducted for seven preoperative parameters: WBC, creatinine, BUN, LDH, CK-MB, SIRI and onset-to-surgery time. Overall, the inter-marker correlations were weak to moderate in magnitude. SIRI demonstrated modest positive correlations with serum creatinine (r = 0.27, *p* < 0.01) and LDH (r = 0.14, *p* < 0.05), indicating that elevated systemic inflammation may coincide with renal impairment and nonspecific cellular injury in patients with TAAD. Notably, LDH also showed weak yet statistically significant associations with WBC (r = 0.23, *p* < 0.01) and CK-MB (r = 0.20, *p* < 0.01), which is consistent with its role as a broad indicator of tissue damage. Onset-to-surgery time showed weak correlations with all preoperative laboratory parameters, with a modest inverse association observed for WBC (r = −0.31, *p* < 0.01). Importantly, no strong correlations (r ≥ 0.70) were observed among these variables, suggesting that these biomarkers capture distinct physiological dimensions and provide largely non-overlapping clinical information.

### 3.3. Prognostic Significance of Preoperative Creatinine or SIRI for OS

**Figure 3:**
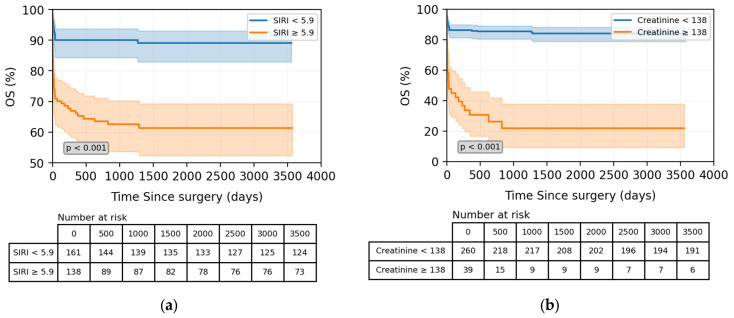
Kaplan-Meier estimate of overall survival in patients according to preoperative SIRI (**a**) and serum creatinine level (**b**).

The TAAD patients with a high SIRI level (≥5.9) had a significantly decreased overall survival than those with a low SIRI level (*p* < 0.001, Figure 3a). Similarly, preoperative high serum creatinine level was associated with decreased OS in TAAD patients (*p* < 0.001, Figure 3b). As shown in the number-at-risk tables, the number of patients at risk decreased gradually over the follow-up period, particularly in the high-risk groups.

### 3.4. Prognostic Significance of Preoperative SCr Score for OS

**Figure 4:**
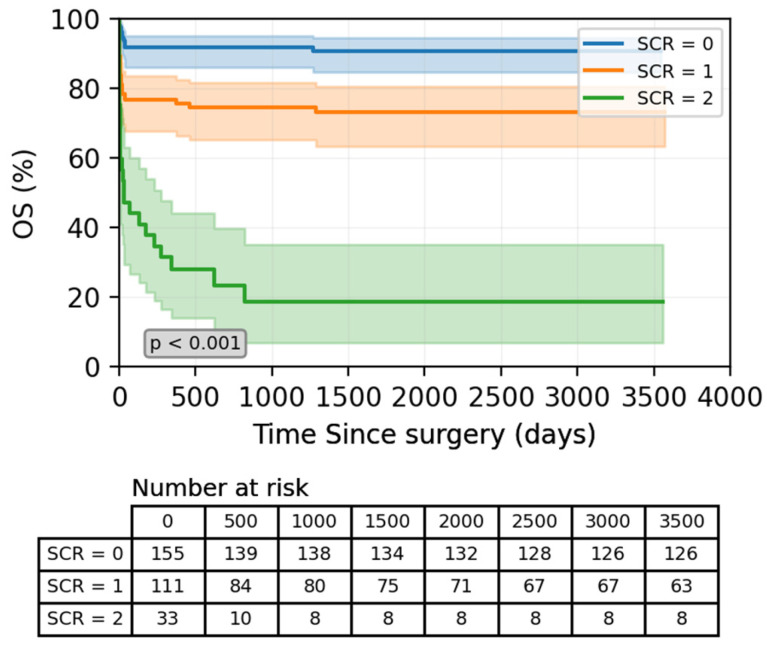
Kaplan–Meier estimate of patients’ overall survival according to the SCr score.

In univariate Cox regression analysis, patients with SCr = 0 served as the reference group. Both elevated SCr scores were associated with progressively higher mortality risks. Compared with the reference group, SCr = 1 showed a moderate increase in risk (HR: 1.353; 95% CI: 1.140–2.187; *p* = 0.018); in comparison, SCr = 2 demonstrated a markedly increased hazard (HR: 7.583; 95% CI: 4.600–12.518; *p* < 0.001, Table 3). Kaplan–Meier survival curves further confirmed this graded risk pattern, with significantly poorer survival in patients with a higher SCr score (Figure 4). These associations still persisted after adjustment for confounders. In the multivariate model, SCr = 1 remained independently associated with increased mortality (HR: 1.876; 95% CI: 1.242–3.733; *p* = 0.013), while SCr = 2 continued to show a strong association (HR: 4.408; 95% CI: 1.786–10.881; *p* = 0.001). Thus, SCr score was confirmed as an independent predictor of long-term survival in TAAD patients (Table 3).

Several additional factors were also identified as independent prognostic indicators in the multivariate Cox model (Table 3). Intraoperative blood loss ≥ 780 mL was associated with significantly increased mortality (HR: 2.473; 95% CI: 1.274–4.801; *p* = 0.007). Prolonged CPB time (≥321 min) was significantly associated with reduced OS (HR: 2.641; 95% CI: 1.455–4.796; *p* = 0.001), and the presence of pleural effusion was also independently associated with elevated mortality (HR: 2.006; 95% CI: 1.270–3.150; *p* = 0.003). Elevated LDH (≥470 U/L) remained a strong predictor of poor outcome (HR: 2.789; 95% CI: 1.495–5.205; *p* = 0.001). Notably, surgical approach (0 * vs. 1 */2 *) emerged as a robust independent predictor of mortality (HR: 4.924; 95% CI: 2.314–10.479; *p* < 0.001). Furthermore, delayed onset-to-surgery time (≥6 h) was independently associated with poorer survival outcomes (HR: 3.065; 95% CI: 1.708–5.502; *p* < 0.001). In contrast, age, shock at admission, renal artery involvement on CTA, stroke, BUN, and CK-MB did not retain independent prognostic significance in the multivariable analysis (Table 3).

### 3.5. Prognostic Significance of Preoperative SCr for IHM

In univariate logistic regression, preoperative SCr score emerged as a significant predictor of IHM in the TAAD cohort (Table 4). Using SCr = 0 as the reference group, patients with SCr = 1 had elevated odds of IHM (OR: 1.719, 95% CI: 1.031–3.173, *p* = 0.033); in comparison, those with SCr = 2 exhibited a markedly elevated risk (OR: 8.036, 95% CI: 3.669–17.604, *p* < 0.001). The significant associations persisted even after controlling for confounders. In the multivariate model, both SCr = 1 (OR: 3.066, 95% CI: 1.032–9.102, *p* = 0.044) and SCr = 2 (OR: 4.811, 95% CI: 1.081–21.409, *p* = 0.039) were independently linked to IHM, confirming that the elevated SCr score represent a strong and graded predictor of postoperative mortality. Several additional factors also retained independent associations with IHM (Table 4). Prolonged CPB time (≥321 min) was strongly associated with increased odds of IHM (OR: 4.868, 95% CI: 1.709–13.866, *p* = 0.003). Intraoperative blood loss ≥ 780 mL was similarly linked to higher mortality risk (OR: 3.049, 95% CI: 1.069–8.697, *p* = 0.037). Pleural effusion emerged as one of the most powerful predictors in the model (OR: 7.918, 95% CI: 2.758–22.732, *p* < 0.001). Elevated LDH levels (≥470 U/L) also remained independently associated with IHM (OR: 5.420, 95% CI: 2.002–14.672, *p* < 0.001), underscoring the prognostic relevance of systemic tissue injury.

In addition, surgical approach showed a strong and independent association with early mortality, with patients undergoing Bentall procedure compared with non-Bentall procedures exhibiting substantially increased odds of IHM (OR: 11.466, 95% CI: 3.088–42.578, *p* < 0.001). Delayed surgical intervention was likewise a significant determinant of outcome, as an onset-to-surgery time ≥ 6 h was associated with a more than tenfold increase in IHM risk (OR: 10.228, 95% CI: 3.582–39.205, *p* < 0.001). In contrast, shock at admission, BUN, CK-MB, renal artery involvement on CTA, and postoperative stroke did not remain significant in the multivariate model, despite demonstrating associations in univariate analysis, suggesting that their prognostic influence may be mediated through other perioperative factors.

**Figure 5:**
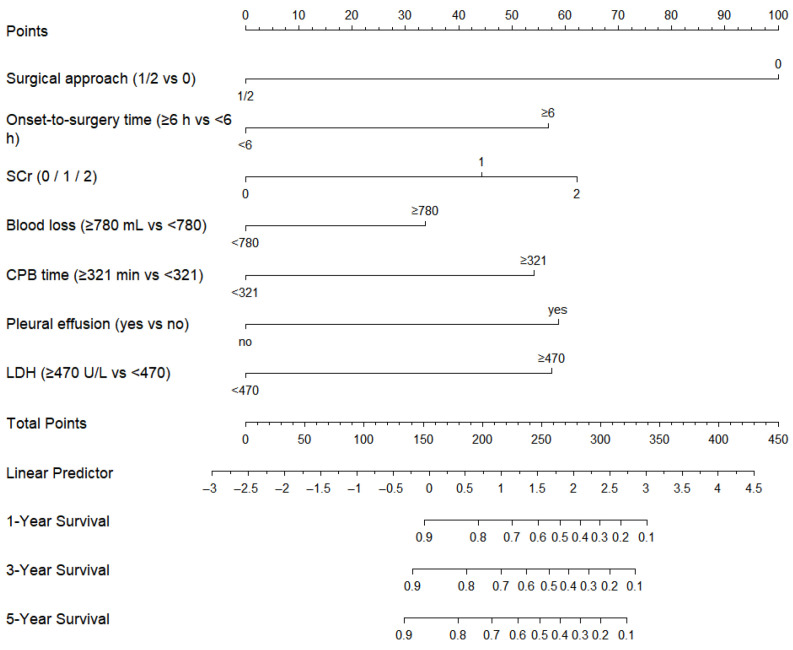
Nomogram for predicting 1-, 3-, and 5-year overall survival in patients with TAAD, incorporating surgical approach, onset-to-surgery time, SCr, blood loss, CPB time, pleural effusion and LDH. Note: ‘Surgical approach’ 0 indicates Bentall procedure; 1 indicates Wheat procedure; 2 indicates ascending aorta replacement.

**Figure 6:**
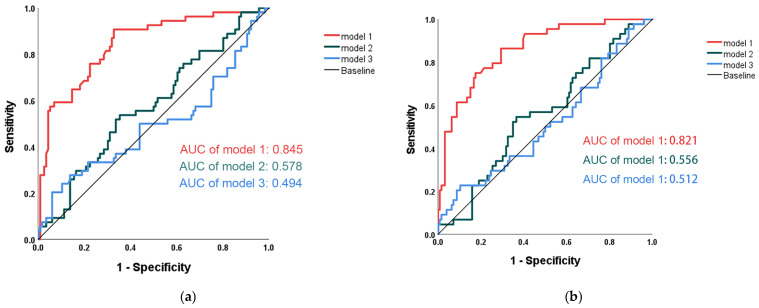
Comparison of predictive ability for OS (**a**) and IHM (**b**) of the AUC between three models for patients with TAAD.

A prognostic nomogram (Model 1) was subsequently developed based on the identified independent factors—surgical approach, onset-to-surgery time, SCr, blood loss, CPB time, pleural effusion and LDH—to estimate overall survival at the 1-, 3-, and 5-year timepoints (Figure 5). Model 1 demonstrated superior predictive performance compared with published models for aortic dissection: Model 2 (uric acid + age + D-dimer) [17] and Model 3 (tenascin-C + D-dimer) [18]. It achieved an AUC of 0.845 (95% CI: 0.781–0.910) for OS, and outperformed Model 2 (AUC: 0.578) and Model 3 (AUC: 0.494) (Figure 6a). Similarly, Model 1 also showed higher predictive accuracy for IHM, with an AUC of 0.821 (95% CI: 0.752–0.891) compared to 0.556 for Model 2 and 0.512 for Model 3 (Figure 6b).

## 4. Discussion

In this study, we propose a composite indicator, the SCr score, which integrates the SIRI and serum creatinine to encompass systemic inflammation, immune activation, and renal dysfunction in patients with TAAD. Among available biomarkers, few provide a unified assessment of both inflammatory burden and organ-level consequences. Our findings demonstrate that an elevated preoperative SCr score was significantly associated with poorer clinical outcomes and independently predicts both short-term and long-term mortality. As an accessible biomarker, the SCr score may provide additional information to assist in the early identification of high-risk patients with TAAD and support perioperative risk stratification within established surgical indication and risk assessment frameworks.

Inflammation is a fundamental driver of aortic dissection pathophysiology. The infiltration of neutrophils and monocytes, combined with cytokine release, accelerates extracellular matrix degradation and propagates aortic injury [19]. Targeted intervention against the inflammatory response may provide new preventive and therapeutic strategies. For example, ulinastatin, a protease inhibitor, possesses anti-inflammatory properties that may attenuate the systemic inflammatory response and reduce organ dysfunction following TAAD surgery [20]. Moreover, an increasing number of systemic inflammation-related scores, such as the NLR, PLR, and SIRI, are being applied to predict postoperative outcomes in patients undergoing surgical intervention for TAAD [21]. SIRI, initially introduced by Qi et al. as a prognostic marker in pancreatic cancer [22], has since demonstrated predictive value across multiple malignancies [23,24,25] and, more recently, cardiovascular diseases [26,27]. The results of large cohort studies have shown that elevated SIRI predicts in-hospital and long-term mortality in heart failure [28], in addition to long-term risks of myocardial infarction and stroke [29]. However, evidence linking SIRI to TAAD remains scarce. The results of prior studies suggest associations between higher SIRI and in-hospital mortality or postoperative complications [6,7], yet its long-term prognostic significance had not been fully clarified prior to this analysis.

Aortic dissection can cause organ malperfusion and fatal vascular rupture. In addition, it can trigger a noninfectious systemic inflammatory response syndrome (SIRS) through mechanisms such as vascular inflammation, macrophage activation, and oxidative stress [30,31]. The excessive secretion of cytokines may further lead to coagulation dysfunction and necroptosis, which can eventually progress to multiple organ dysfunction syndrome (MODS) [30]. Acute kidney injury (AKI) is common in patients with aortic dissection. Factors such as dissection tear-induced renal hypoperfusion, inflammatory biomarker-mediated vascular injury, and pre-existing chronic kidney disease may indirectly increase the risk of preoperative AKI [32,33]. In contrast, postoperative AKI is more significantly influenced by the type of vascular surgery performed, the inflammatory response, CPB time, and preoperative renal function [33]. Elevated serum creatinine often reflects renal malperfusion or inflammation-mediated renal injury, and it serves as a well-established clinical indicator for renal perfusion and systemic injury in cardiovascular diseases, including TAAD and thoracic aortic aneurysms [34,35,36,37]. Although elevated serum creatinine levels have been linked to early mortality or postoperative complications in some studies [9,38,39], findings on long-term outcomes have been inconsistent. In our cohort, elevated preoperative serum creatinine was consistently associated with a poorer prognosis, reinforcing its relevance as an adverse risk indicator.

By examining the SIRI and serum creatinine separately, we observed that both were associated with worse survival. In fact, serum creatinine levels are not a highly sensitive indicator of renal function, as they do not show significant elevation until the glomerular filtration rate declines by more than 50% [40]. Building on this foundation, we constructed a composite index, the SCr score, to simultaneously reflect upstream inflammatory activity and downstream renal dysfunction. The result of multivariable analyses confirmed the SCr score as an independent predictor of both OS and IHM, with graded increases in risk across the three SCr scores. The biological rationale for integrating the SIRI and serum creatinine lies in the interconnected roles of inflammation and renal injury in TAAD. Systemic inflammation intensifies aortic wall degeneration and affects multiple organs, and renal impairment further amplifies hemodynamic instability and complicates postoperative recovery [12,13,41]. The SCr score captures these complementary dimensions, providing a clinically practical composite measure of physiological stress. Our results show that the SCr score demonstrates strong and consistent associations with both early and late mortality, supporting its potential utility for perioperative risk stratification.

Using the SCr score as an important predictor, we developed a prognostic nomogram that incorporated additional independent risk factors in our study. Surgical approach, onset-to-surgery time, blood loss, CPB time, pleural effusion and LDH were also independently associated with long-term mortality—a finding consistent with known contributors to adverse outcomes in TAAD [11,14]. The interval from symptom onset to surgical intervention has been reported as an important prognostic factor in acute aortic dissection. Consistent with prior reports, our findings demonstrate that a prolonged onset-to-surgery time (≥6 h) was independently associated with increased long-term mortality. Given the severity of disease in patients treated at our center, irreversible aortic root damage was common, and the Bentall procedure was therefore the predominant surgical strategy. Surgical approach thus reflected disease extent and operative complexity and was independently associated with long-term mortality. All procedures were performed under emergency conditions rather than electively, which may partly explain the prolonged CPB duration and increased intraoperative blood loss observed in this cohort, potentially contributing to adverse outcomes through intensified ischemia–reperfusion injury and systemic inflammation. Pleural effusion, potentially caused by hemodynamic instability or an inflammatory reaction, can aggravate respiratory failure or signal dissection expansion [42]. In AD, acute ischemia and inflammation of the aortic wall can lead to extensive cellular damage and the release of LDH into the blood, indicating substantial tissue injury, which indicates substantial tissue damage [43]. Our model based on these risk factors achieved higher discrimination for OS (AUC: 0.845) and IHM (AUC: 0.821), outperforming two previously published models in aortic dissection [17,18]. These findings suggest that SCr-based risk assessment may assist in early perioperative risk stratification in patients with TAAD and support more individualized perioperative management strategies, including intensified monitoring and organ-protective measures. However, the SCr-based index should be interpreted as a complementary risk marker within a comprehensive clinical framework, rather than as a standalone determinant of prognosis.

Several limitations merit consideration. The first limitation of this study is its single-institution retrospective design; as such, selection bias cannot be entirely ruled out. Second, although the sample size was sufficient for model development, external validation in larger, multi-center cohorts is necessary. Third, although perioperative echocardiographic parameters (e.g., left ventricular ejection fraction and valvular function) are relevant to prognosis, these data were not available in this retrospective study and thus were not included in the analysis. Fourth, although renal artery involvement on CTA and proximal repair strategy were included in the analyses, more detailed anatomical and intraoperative variables (e.g., branch-vessel malperfusion extent, tear characteristics, and procedural variations) were not available for adjustment. Residual confounding from these unmeasured factors may have influenced the observed associations. Finally, although the SCr score provides prognostic information, it is not intended to inform decisions regarding surgical indication, which should continue to be guided by established assessment frameworks. Instead, the clinical value of SCr-based risk stratification lies in its ability to identify patients at increased risk of adverse outcomes, thereby enabling more individualized perioperative management.

## 5. Conclusions

This study demonstrates that an elevated preoperative SCr score is independently associated with increased in-hospital mortality and worse long-term survival among patients with DeBakey type I Stanford type A aortic dissection undergoing surgical repair, after adjustment for key prognostic factors including surgical approach, onset-to-surgery time, CPB time, and renal artery involvement on CTA. By focusing on a homogeneous cohort of DeBakey type I dissections, the proposed prognostic nomogram achieved improved discriminative performance for risk stratification. While more granular anatomical heterogeneity within DeBakey type I TAAD may further influence outcomes and warrants future investigation, SCr-based assessment may support refined perioperative risk evaluation and individualized clinical management.

## Figures and Tables

**Table 1:** SCr score system.

SIRI	Creatinine (μmol/L)	SCr Score
<5.9	<138	0
≥5.9	<138	1
<5.9	≥138	1
≥5.9	≥138	2

**Table 2:** Descriptive summary of patient characteristics stratified by survival status.

Variables	All Patients (n = 299)	Non-Survivors (n = 68)	Survivors (n = 231)
Demographic statistics			
Gender, *n* (%)			
Female	76 (25.4%)	18 (26.5%)	58 (25.1%)
Male	223 (74.6%)	50 (73.5%)	173 (74.9%)
Age (years)	50.2 ± 9.2	51.2 ± 10.2	49.9 ± 11.8
Risk factors, *n* (%)			
Smoking			
No	109 (36.5%)	31 (45.6%)	78 (33.8%)
Yes	190 (63.5%)	37 (54.4%)	153 (66.2%)
Alcohol use			
No	124 (41.5%)	28 (41.2%)	96 (41.6%)
Yes	175 (58.5%)	40 (58.8%)	135 (58.4%)
BMI (Kg/m^2^)	25.3 ± 2.5	25.2 ± 3.0	25.4 ± 2.4
HTN			
No	94 (31.4%)	21 (30.9%)	73 (31.6%)
Yes	205 (68.6%)	47 (69.1%)	158 (68.4%)
DM			
No	295 (98.7%)	65 (95.6%)	230 (99.6%)
Yes	4 (1.3%)	3 (4.4%)	1 (0.4%)
Status at admission			
SBP (mmHg)	145.4 ± 25.5	146.9 ± 23.3	140.3 ± 31.4
DBP (mmHg)	85.3 ± 16.6	86.4 ± 15.0	81.6 ± 20.9
Shock, *n* (%)			
No	281 (94.0%)	53 (77.9%)	228 (98.7%)
Yes	18 (6.0%)	15 (22.1%)	3 (1.3%)
Heart rate (b.p.m.)	83.7 ± 12.6	84.9 ± 13.9	83.3 ± 12.2
Chest pain, *n* (%)			
No	27 (9.0%)	11 (16.2%)	16 (6.9%)
Yes	272 (91.0%)	57 (83.8%)	215 (93.1%)
Back pain, *n* (%)			
No	73 (24.4%)	23 (33.8%)	50 (21.6%)
Yes	226 (75.6%)	45 (66.2%)	181 (78.4%)
Abdominal pain, *n* (%)			
No	275 (92.0%)	50 (73.5%)	225 (97.4%)
Yes	24 (8.0%)	18 (26.5%)	6 (2.6%)
Altered consciousness, *n* (%)			
No	284 (95.0%)	57 (83.8%)	227 (98.3%)
Yes	15 (5.0%)	11 (16.2%)	4 (1.7%)
ST-segment deviation on ECG, *n* (%)			
No	187 (62.5%)	37 (54.4%)	150 (64.9%)
Yes	111 (37.1%)	30 (44.1%)	81 (35.1%)
Pericardial effusion, *n* (%)			
No	162 (54.2%)	32 (47.1%)	130 (56.3%)
Yes	137 (45.8%)	36 (52.9%)	101 (43.7%)
Pleural effusion, *n* (%)			
No	171 (57.2%)	22 (32.4%)	149 (64.5%)
Yes	128 (42.8%)	46 (67.6%)	82 (35.5%)
Preoperative clinical, laboratory, and imaging characteristics			
WBC (×10^9^/L)	11.7 ± 3.5	13.7 ± 4.4	11.2 ± 2.9
Hemoglobin (g/L)	125.3 ± 22.5	122.8 ± 24.7	126.0 ± 21.8
Platelet count (×10^9^/L)	164.7 ± 52.3	157.6 ± 56.7	166.8 ± 50.9
Total protein (g/L)	65.4 ± 5.6	64.8 ± 6.2	65.6 ± 5.4
Albumin (g/L)	36.1 ± 8.7	34.1 ± 10.1	36.6 ± 8.2
ALT (U/L)	35.5 ± 94.9	62.4 ± 194.8	27.6 ± 18.4
AST (U/L)	46.2 ± 130.3	87.5 ± 257.2	34.0 ± 45.4
Creatinine (μmol/L)	98.8 ± 55.9	145.0 ± 93.0	85.2 ± 26.7
Uric acid (μmol/L)	262.6 ± 173.5	269.6 ± 232.7	260.5 ± 152.3
BUN (mmol/L)	11.3 ± 13.5	14.3 ± 13.7	10.4 ± 13.3
LDH (U/L)	567.4 ± 745.1	866.2 ± 1438.4	479.4 ± 287.1
D-dimer (mg/L)	9.4 ± 14.5	11.7 ± 17.2	8.7 ± 13.5
Fibrinogen (g/L)	2.9 ± 1.4	3.2 ± 1.9	2.8 ± 1.3
FDP (μg/mL)	30.0 ± 67.2	45.7 ± 86.9	25.4 ± 59.6
Myoglobin (μg/L)	134.5 ± 376.6	179.6 ± 273.1	121.3 ± 401.6
CK-MB (μg/L)	3.8 ± 6.9	6.3 ± 9.5	3.1 ± 5.8
BNP (pg/mL)	973.5 ± 1869.9	1067.7 ± 1992.5	945.8 ± 1835.8
SIRI	9.9 ± 14.0	15.7 ± 19.1	8.2 ± 11.5
Renal artery involvement on CTA, *n* (%)			
No	229 (76.6%)	45 (66.2%)	184 (79.65%)
Yes	70 (23.4%)	23 (33.8%)	47 (20.35%)
Onset-to-surgery time (hours)	21.7 ± 21.3	24.8 ± 23.4	16.5 ± 12.3
Intraoperative conditions			
Operation time (minutes)	529.2 ± 118.0	626.4 ± 153.3	500.6 ± 87.1
Intraoperative blood loss(mL)	664.3 ± 651.5	1161.8 ± 1100.3	517.8 ± 320.4
Transfusion of SRBC (U)	3.4 ± 3.2	6.0 ± 4.7	2.7 ± 2.1
CPB time (min)	255.9 ± 70.1	314.5 ± 86.9	238.6 ± 53.4
ACC time (min)	153.5 ± 41.6	176.9 ± 42.6	146.6 ± 38.8
Surgical approach, *n* (%) *			
Bentall procedure	185 (61.9%)	59 (86.8%)	126 (54.55%)
Wheat procedure	104 (34.8%)	8 (11.8%)	96 (41.56%)
Ascending aorta replacement	10 (3.3%)	1 (1.5%)	9 (3.90%)
Pacemaker implantation, *n* (%)			
No	180 (60.2%)	35 (51.5%)	145 (62.8%)
Yes	119 (39.8%)	33 (48.5%)	86 (37.2%)
Postoperative outcomes			
Dialysis, *n* (%)			
No	209 (69.9%)	29 (42.6%)	180 (77.9%)
Yes	90 (30.1%)	39 (57.4%)	51 (22.1%)
Stroke, *n* (%)			
No	262 (87.6%)	51 (75.0%)	211 (91.3%)
Yes	37 (12.4%)	17 (25.0%)	20 (8.7%)

BMI, body mass index; HTN, hypertension; DM, diabetes mellitus; SBP, systolic blood pressure; DBP, diastolic blood pressure; b.p.m., beats per minute; ECG, electrocardiogram; WBC, white blood cell count; ALT, alanine aminotransferase; AST, aspartate aminotransferase; BUN, blood urea nitrogen; LDH, lactate dehydrogenase; FDP, fibrin degradation products; CK-MB, creatine kinase-muscle/brain; BNP, B-type natriuretic peptide; SIRI, systemic inflammation response index. CTA, computed tomography angiography; SRBC, suspended red blood cell; CPB, cardiopulmonary bypass; ACC, aortic cross-clamp. * Note on surgical approach: All patients included in this cohort underwent total aortic arch replacement combined with frozen elephant trunk implantation. The listed surgical approaches (Bentall procedure, Wheat procedure, ascending aortic replacement) refer to surgical procedures for proximal aortic/root reconstruction.

**Table 3:** The effect of clinical variables and SCr score on OS.

Variables	Univariate Analysis		Multivariate Analysis	
	HR (95% CI)	*p*	HR (95% CI)	*p*
Age (years, <52 vs. ≥52)	0.792 (0.500–1.274)	0.335	1.467 (0.763–2.820)	0.251
Shock (yes vs. no)	6.798 (3.150–12.140)	<0.001	1.503 (0.659–3.428)	0.333
SIRI (<5.9 vs. ≥ 5.9)	4.176 (2.410–7.235)	<0.001		NI
Creatinine (μmol/L, <138 vs. ≥138)	7.623 (4.670–12.456)	<0.001		NI
SCr (0)		Ref		Ref
SCr (1)	1.353 (1.140–2.187)	0.018	1.876 (1.242–3.733)	0.013
SCr (2)	7.583 (4.600–12.518)	<0.001	4.408 (1.786–10.881)	0.001
Blood loss (ml, ≥780 vs. <780)	7.690 (4.730–12.480)	<0.001	2.473 (1.274–4.801)	0.007
CPB time (minutes, ≥321 vs. <321)	5.769 (3.490–9.526)	<0.001	2.641 (1.455–4.796)	0.001
Pleural effusion (yes vs. no)	3.153 (1.910–5.242)	0.002	2.006 (1.270–3.150)	0.003
BUN (mmol/L, ≥18.3 vs. <18.3)	3.424 (2.010–5.959)	<0.001	0.233 (0.082–0.661)	0.796
CK-MB (μg/L, ≥8.6 vs. <8.6)	4.119 (2.150–7.890)	0.027	0.644 (0.283–1.462)	0.293
LDH (U/L, ≥470 vs. <470)	4.450 (2.670–7.503)	<0.001	2.789 (1.495–5.205)	0.001
Renal artery involvement on CTA (yes vs. no)	1.899 (1.148–3.141)	0.013	1.198 (0.568–2.528)	0.636
Stroke (yes vs. no)	3.154 (1.815–5.480)	<0.001	1.407 (0.606–3.269)	0.427
Surgical approach (0 * vs. 1 */2 *)	4.963 (2.458–10.021)	<0.001	4.924 (2.314–10.479)	<0.001
Onset-to-surgery time (hours, ≥6 vs. <6)	4.279 (2.653–6.903)	<0.001	3.065 (1.708–5.502)	<0.001

HR, hazard ratio; NI, not included; 0 * indicates Bentall procedure; 1 * indicates Wheat procedure; 2 * indicates ascending aorta replacement. SIRI and creatinine were not included in the multivariate model as they were integrated into the composite SCr index.

**Table 4:** The effect of clinical variables and SCr score on IHM.

Variables	Univariate Analysis		Multivariate Analysis	
	OR (95% CI)	*p*	OR (95% CI)	*p*
Age (years, <52 vs. ≥52)	0.469 (0.252–0.876)	0.118	2.400 (0.784–7.351)	0.125
Shock (yes vs. no)	9.751 (3.565–26.670)	0.003	2.690 (0.583–12.414)	0.205
SIRI (<5.9 vs. ≥5.9)	4.718 (2.351–9.470)	<0.001		NI
Creatinine (μmol/L, <138 vs. ≥138)	8.556 (4.075–17.963)	<0.001		NI
SCr (0)		Ref		Ref
SCr (1)	1.719 (1.031–3.173)	0.033	3.066 (1.032–9.102)	0.044
SCr (2)	8.036 (3.669–17.604)	<0.001	4.811 (1.081–21.409)	0.039
Blood loss (ml, ≥780 vs. <780)	8.531 (4.172–17.443)	<0.001	3.049 (1.069–8.697)	0.037
CPB time (minutes, ≥321 vs. <321)	10.083 (4.830–21.052)	0.029	4.868 (1.709–13.866)	0.003
Pleural effusion (yes vs. no)	3.147 (1.662–5.959)	0.011	7.918 (2.758–22.732)	<0.001
BUN (mmol/L, ≥18.3 vs. <18.3)	2.265 (0.974–5.267)	0.058	0.312 (0.053–1.843)	0.199
CK-MB (μg/L, ≥8.6 vs. <8.6)	8.497 (3.216–22.448)	0.004	2.624 (0.616–11.170)	0.192
LDH (U/L, ≥470 vs. <470)	7.221 (3.517–14.826)	<0.001	5.420 (2.002–14.672)	<0.001
Renal artery involvement on CTA (yes vs. no)	1.905 (0.986–3.683)	0.055	0.583 (0.126–2.694)	0.490
Stroke (yes vs. no)	3.821 (1.802–8.104)	<0.001	2.275 (0.436–11.868)	0.329
Surgical approach (0 * vs. 1 */2 *)	7.007 (2.690–18.250)	<0.001	11.466 (3.088–42.578)	<0.001
Onset-to-surgery time (hours, ≥6 vs. <6)	5.730 (2.976–11.033)	<0.001	10.228 (3.582–39.205)	<0.001

OR, odds ratio; NI, not included; 0 * indicates Bentall procedure; 1 * indicates Wheat procedure; 2 * indicates ascending aorta replacement. SIRI and creatinine were not included in the multivariate model as they were integrated into the composite SCr index.

## Data Availability

The data supporting this article are available from the corresponding author upon reasonable request.

## Supplementary Materials

The following supporting information can be downloaded at: https://www.mdpi.com/article/10.3390/jcdd13030133/s1, Figure S1: Histogram distribution and cutoff determination for candidate variables. Panels A–I illustrate the distribution patterns of nine continuous variables and their optimal cutoff values determined using X-tile software. (A) Age (cutoff = 53 years), (B) SIRI (cutoff = 5.9), (C) BUN (cutoff = 18.3 mmol/L), (D) CK-MB (cutoff = 8.6 μg/L), (E) LDH (cutoff = 470 U/L), (F) Serum creatinine (Cr, cutoff = 138 μmol/L), (G) CPB time (cutoff = 321 min), (H) Intraoperative blood loss (cutoff = 780 mL), (I) Onset-to-surgery time (cutoff = 6 h). Table S1. Variance inflation factors (VIFs) for variables included in the multivariable model.

## Abbreviations

The following abbreviations are used in this manuscript:

TAAD	Type A aortic dissection
SIRI	Systemic inflammation response index
OS	Overall survival
IHM	In-hospital mortality
ROC	Receiver operating characteristic
HR	Hazard ratio
CI	Confidence interval
OR	Odds ratio
AUC	Area under the curve
CRP	C-reactive protein
PCT	Procalcitonin
IL-6	Interleukin-6
NLR	Neutrophil-to-lymphocyte ratios
PLR	Platelet-to-lymphocyte ratios
BMI	Body mass index
HTN	Hypertension
DM	Diabetes mellitus
BUN	Blood urea nitrogen
CK-MB	Creatine kinase–muscle/brain
LDH	Lactate dehydrogenase
CPB	Cardiopulmonary bypass
ACC	Aortic cross clamp
SBP	Systolic blood pressure
DBP	Diastolic blood pressure
b.p.m.	Beats per minute
WBC	White blood cell count
ALT	Alanine aminotransferase
AST	Aspartate aminotransferase
FDP	Fibrin degradation products
BNP	B-type natriuretic peptide
SRBC	Suspended red blood cell
SIRS	Systemic inflammatory response syndrome
MODS	Multiple organ dysfunction syndrome
AKI	Acute kidney injury
