# Supplementary material for: Prognostic Value of a Composite Inflammation–Renal Function Score in Type A Aortic Dissection
Source: J Cardiovasc Dev Dis. 2026 Mar 11;13(3):133. doi: 10.3390/jcdd13030133 (PMC13027188; doi:10.3390/jcdd13030133)
Supplement: Supplementary file 1 [file jcdd-13-00133-s001.zip › jcdd-4091164-supplementary-proof.pdf]

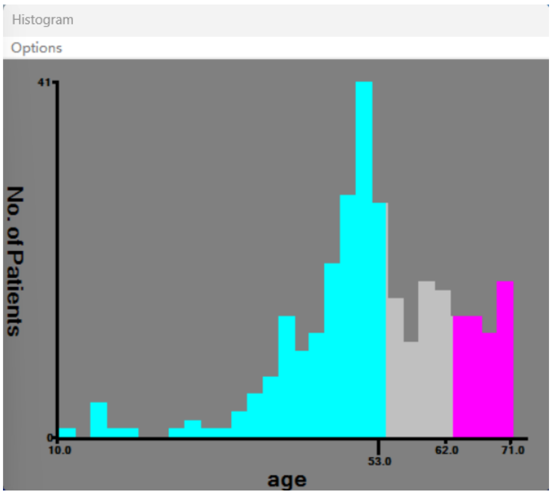
Histogram
Options
No. of Patients
41
0
10.0
53.0
62.0
71.0
age

A

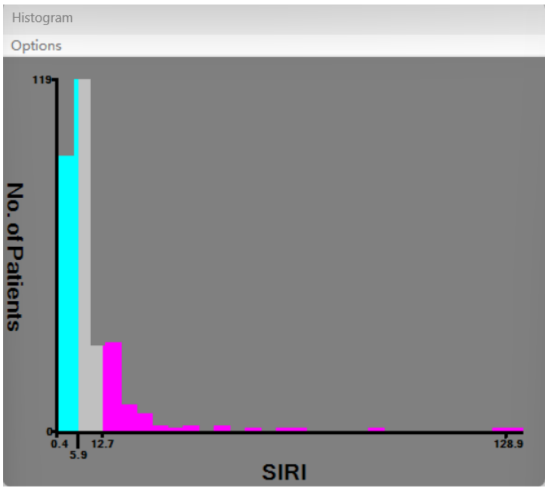
Histogram
Options
No. of Patients
119
0
0.4
5.9
12.7
128.9
SIRI

B

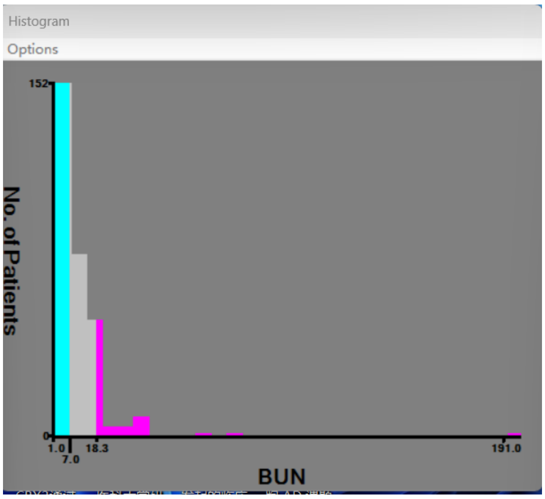
Histogram
Options
No. of Patients
152
0
1.0
7.0
18.3
191.0
BUN

C

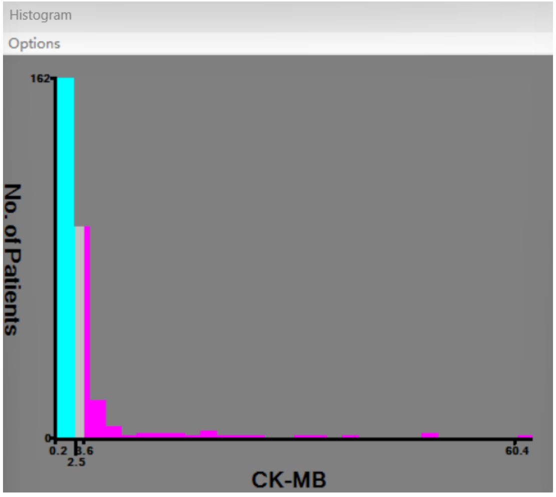
Histogram
Options
No. of Patients
162
0
0.2
2.5
3.6
60.4
CK-MB

D

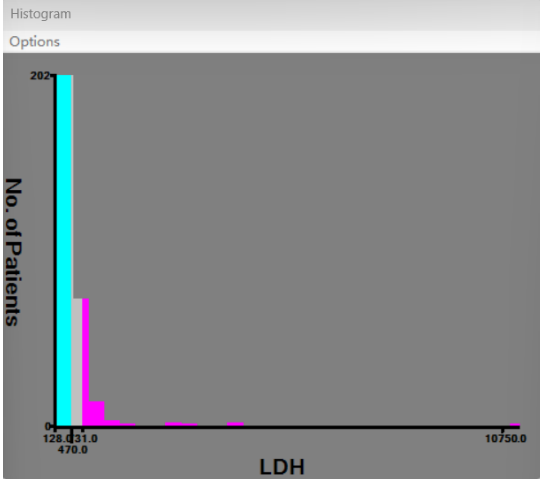
Histogram
Options
No. of Patients
202
0
128.0
470.0
631.0
10750.0
LDH

E

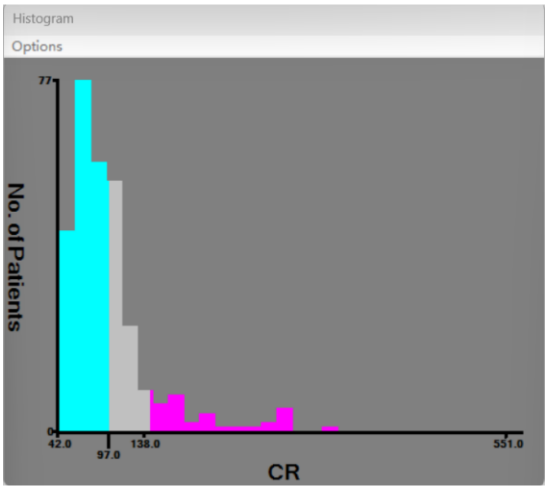
Histogram
Options
No. of Patients
77
0
42.0
97.0
138.0
551.0
CR

F

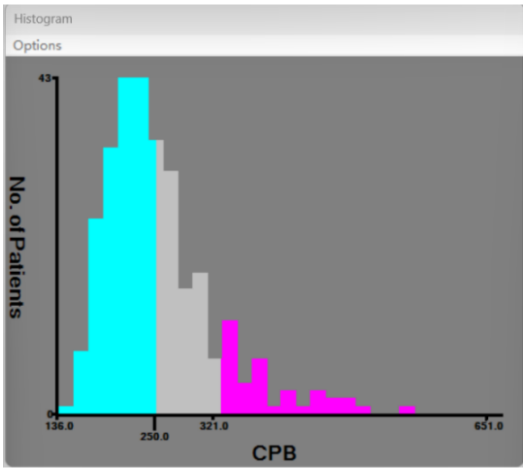

G

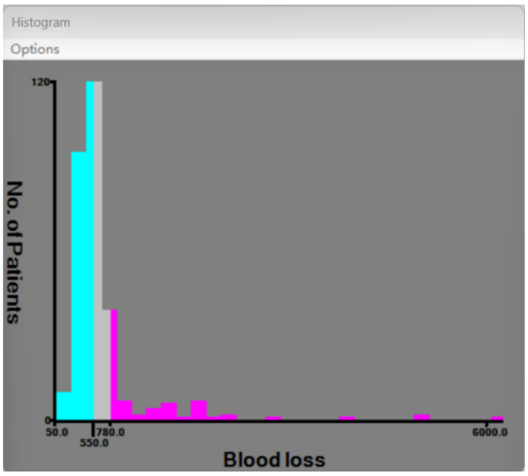

H

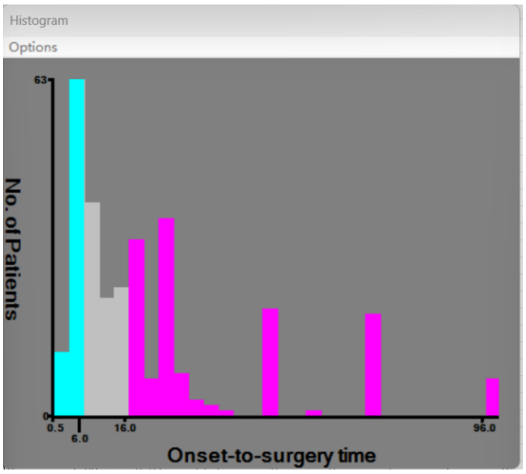

I

Figure S1. Histogram distribution and cutoff determination for candidate variables.

Panels A–I illustrate the distribution patterns of nine continuous variables and their optimal cutoff values determined using X-tile software. (A) Age (cutoff = 53 years), (B) SIRI (cutoff = 5.9), (C) BUN (cutoff = 18.3 mmol/L), (D) CK-MB (cutoff = 8.6 μg/L), (E) LDH (cutoff = 470 U/L), (F) Serum creatinine (Cr, cutoff = 138 μmol/L), (G) CPB time (cutoff = 321 minutes), (H) Intraoperative blood loss (cutoff = 780 mL), (I) Onset-to-surgery time (cutoff = 6 hours).

Table S1. Variance inflation factors (VIF) for variables included in the multivariable model

Variables	VIF
Age	1.84
Shock	1.63
SCr	1.61
Blood loss	1.60
CPB time	1.44
Pleural effusion	1.37
BUN	1.30
CK-MB	1.24
LDH	1.18
Renal artery involvement on CTA	1.16
Stroke	1.14
Surgical approach	1.11
Onset-to-surgery time	1.08
